# Machine learning glass transition temperature of polymers

**DOI:** 10.1016/j.heliyon.2020.e05055

**Published:** 2020-10-06

**Authors:** Yun Zhang, Xiaojie Xu

**Affiliations:** North Carolina State University, Raleigh, NC 27695, USA

**Keywords:** Materials science, Materials chemistry, Physical chemistry, Glass transition temperature, Polymer, Machine learning, Gaussian process regression

## Abstract

As an important thermophysical property, polymers' glass transition temperature, Tg, could sometimes be difficult to determine experimentally. Modeling methods, particularly data-driven approaches, are promising alternatives to predictions of Tg in a fast and robust way. The molecular traceless quadrupole moment and molecule average hexadecapole moment are closely correlated with polymers' Tg. In the current work, these two parameters are used as descriptors in the Gaussian process regression model to predict Tg. We investigate 60 samples with Tg values from 194 K to 440 K. The model provides rapid and low-cost Tg estimations with high accuracy and stability.

## 1 Introduction

Glass transition temperature, Tg, of polymeric materials is a complex parameter that depends on both intrinsic and extrinsic factors. The morphology, crystallinity, tacticity, molecular weight, and density are a few examples of intrinsic factors, while synthesis and characterization methods are examples of extrinsic ones. These factors determine transition temperature of the amorphous phase between glassy and rubbery states, which plays a critical role in the polymer characterization and application [1]. With the change of the state around Tg, other physical properties vary, including the modulus, heat capacities, thermal expansion coefficient, and viscosity. For example, effects of the irradiation dose on Tg values of cyanate ester/epoxy resin utilized as insulation materials for superconducting magnets are studied to evaluate toughening mechanisms of the composite [2], [3], [4], [5], [6]. During the synthesis of half-metallic CrO2/polystyrene composites with enhanced room-temperature magnetoresistance, Tg of polystyrene affects the thermal caloricity behavior of composites and thus performance [7], [8], [9], [10], [11], [12]. As for polymer semiconductors, Tg also impacts the polymer solar cell stability as it governs molecular organization's kinetics during the solidification [13], [14], [15], [16].

Experimentally, Tg of a certain material could be measured and decided by several instrumentations, such as DMA–dynamic mechanical analysis, DSC–differential scanning calorimetry, and TMA–thermomechanical analysis. However, the glass transition step usually spans over a large range of temperature and depends heavily on measurement conditions, including the initial polymer relaxation condition, heating and cooling rates, and the gas atmosphere. To facilitate the design and application of polymers, theoretical calculations and empirical modeling have been used to estimate Tg values. By combining the density function theory, mode coupling, and activated hopping transport theories, a statistical mechanical theory has been built by describing polymer chains as coarse grains and describing melt as liquid of segments [17], [18], [19]. Besides, by modifying the polymer consistent force field, a molecular dynamics simulation based approach has been carried out to predict Tg of polyhydroxyalkanoates-based polymers. Density functional theory is used to refine a set of torsion potentials of the polymer backbone, including the number of polymer chains, chain length, supercell size, and simulated thermal quenching rate [20], [21], [22], [23], [24], [25]. The effective activation energy and mutual diffusion coefficient is dependent on temperature, as shown in the enhanced model describing the polymer-solvent diffusion above and below Tg [26]. However, a significant amount of descriptor calculations and data input is required by most of these models, along with the need of heavy computational resources and the optimization of models. To provide fast, simple, and low-cost estimations of polymer Tg, data-driven approaches based on readily-available physiochemical parameters have been promising alternatives to conventional simulation tools. These approaches create surrogate models utilizing machine learning techniques, which aim at presenting rapid predictions of critical materials performance target values.

We build the GPR–Gaussian process regression model for shedding light upon relationships among the molecular traceless quadrupole moment (Θ), the molecular average hexadecapole moment (Φ), and glass transition temperature for polymers. The model generalizes well with the capability of pattern learning and recognition. It manifests high accuracy and stability and thus makes contributions to estimating glass transition temperature efficiently at low cost and helps its understanding from Θ and Φ. GPRs have been employed in many materials systems for predicting different parameters in a wide variety of fields [27], [28], [29], [30], [31], [32], [33], [34], [35], [36], [37], [38], [39], [40], [41], [42], [43], [44], [45], [46], [47]. Our model could be adopted as a guideline to design polymers and might be utilized to facilitate understandings of relationships between fundamental parameters and glass transition temperature.

## 2 Methodology

**Figure 1:**
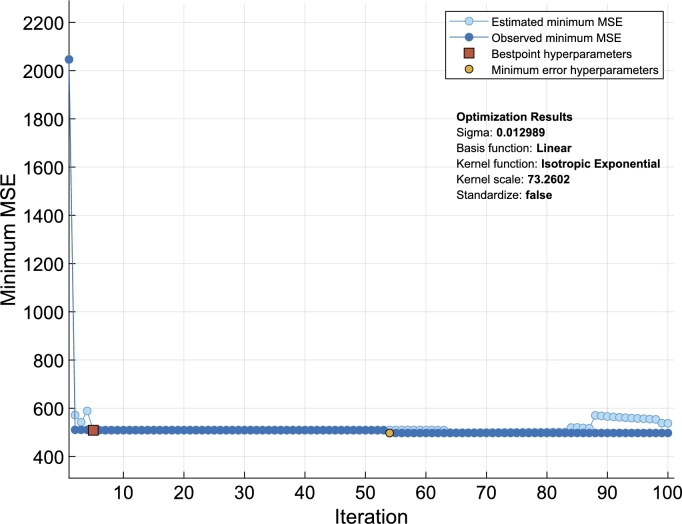
Bayesian optimizations. The process involves the ten kernels and four basis functions mentioned in Section 2, and arrives at the final specification of the isotropic exponential kernel and the linear basis with independent variables not standardized. *σ* is used as the initial value for developing Models M1, M2.CV F1, M2.CV F2, ..., and M2.CV F10 (see Table 2 for specifications).

GPRs belong to nonparametric probabilistic models that are kernel-based. We explore five isotropic kernels (Rational Quadratic, Matern 5/2, Matern 3/2, Squared Exponential, and Exponential) and their five associated non-isotropic kernels. We examined four basis functions (Pure Quadratic, Empty, Linear, and Constant). We employ cross validation and LCB (lower confidence bound) Bayesian optimizations for estimating parameters (Fig. 1). We use the mean absolute error (MAE), together with the root mean square error (RMSE), and correlation coefficient (CC) for performance assessments. More technical details can be found from [27], [28], [29], [30], [31], [32], [33], [34], [35], [36], [37], [38], [40], [42], [43], [44], [45], [47].

## 3 Data

**Figure 2:**
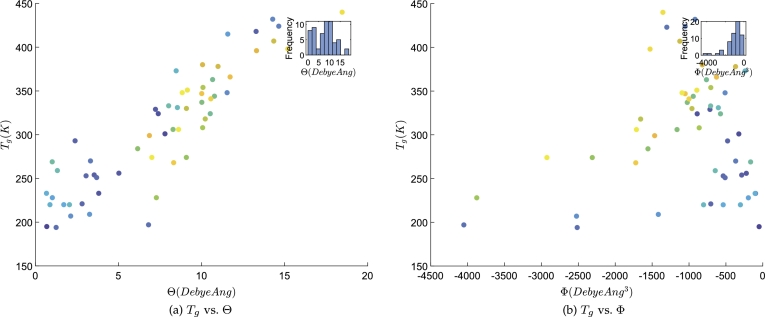
Data visualization. Θ–the molecular traceless quadrupole moment and Φ–the molecular average hexadecapole moment serve as descriptors for polymers. *T*_*g*_(*K*)–glass transition temperature serves as the target variable. (a) *T*_*g*_ vs. Θ plots the glass transition temperature and molecular traceless quadrupole moment. (b) *T*_*g*_ vs. Φ plots the glass transition temperature and molecular average hexadecapole moment.

Data listed in Columns 2–5 of Table 1 are collected from [48], [49], covering a wide variety of polymers. 60 polymers with glass transition temperature from 194 K to 440 K are investigated. Θ–the molecular traceless quadrupole moment and Φ–the molecular average hexadecapole moment serve as descriptors. Data are plotted in Fig. 2, which suggests nonlinear patterns that we use the GPR to model.

**Table 1:** Data for modeling and predictions.

Index	Polymer	Θ (Debye Ang)	Φ (Debye Ang^3^)	Tg (K)	QSPR	SVR	M1	CV Fold	M2. CV Fi	M2
1	Polyethylene	0.6776	−50.0178	195	221	222	195.0366	CV F3	234.2390	198.9571
2	Poly (vinyl acetate)	7.7949	−324.9730	301	324	321	301.0246	CV F1	325.0341	303.4257
3	Poly (vinyl butyral)	7.3902	−889.1739	324	305	306	323.9881	CV F3	315.2997	323.1181
4	Poly (vinylidene fluoride)	3.8068	−98.8344	233	268	265	233.0538	CV F5	273.9645	237.1441
5	Poly (vinylidene chloride)	5.0194	−223.2520	256	283	281	256.0370	CV F6	291.9063	259.6241
6	Poly (vinyl isobutyral)	7.2233	−716.3426	329	306	306	328.9774	CV F7	306.6759	326.7474
7	Poly (vinyl ethyl ether)	3.5334	−286.2433	254	259	258	254.0111	CV F6	264.4267	255.0530
8	Poly (N-vinyl pyrrolidone)	13.2808	−458.8428	418	405	396	417.9862	CV F4	404.5722	416.6441
9	Poly (vinyl propionate)	2.3829	−477.3837	293	237	239	292.9571	CV F7	245.3008	288.1910
10	Poly (vinyl n-butyl ether)	2.8052	−704.9683	221	238	241	221.0424	CV F2	255.2825	224.4639
11	Poly (vinyl sec-butyl ether)	3.0442	−536.6563	253	246	247	252.9601	CV F5	240.6045	251.7294
12	Poly (vinyl isobutyl ether)	3.6811	−510.9593	251	256	257	251.0033	CV F1	254.8251	251.3870
13	Poly (vinyl n-octyl ether)	1.2418	−2515.0488	194	174	191	193.9905	CV F6	185.0638	193.0971
14	Poly (vinyl n-decyl ether)	6.8052	−4050.9702	197	224	232	197.0310	CV F1	229.4064	200.2681
15	Poly (N-vinyl carbazole)	17.5803	−1301.0913	423	452	429	423.0194	CV F2	438.0238	424.5206
16	Poly (vinyl isopropyl ether)	3.3098	−367.8203	270	254	254	269.9918	CV F4	259.3943	268.9320
17	Poly (b-vinyl naphthalene)	14.6455	−1051.8587	424	413	403	423.9786	CV F8	405.9218	422.1732
18	Poly (a-vinyl naphthalene)	14.2807	−918.0995	432	410	401	431.9701	CV F9	403.0533	429.0783
19	Poly (vinyl chloroacetate)	11.5354	−511.4806	348	377	372	348.0262	CV F9	369.3863	350.1629
20	Poly (vinyl 2-ethylhexyl ether)	2.1186	−2524.2913	207	187	201	206.9894	CV F3	192.3947	205.5297
21	Poly (vinyl n-hexyl ether)	3.2550	−1415.7591	209	229	236	209.0280	CV F9	237.9892	211.9242
22	Poly (p-vinyl pyridine)	11.5756	−474.2108	415	379	374	414.9705	CV F8	378.3183	411.3038
23	Poly (ethylethylene)	1.0142	−195.7518	228	222	224	228.0034	CV F2	232.2203	228.4253
24	Poly (butylethylene)	1.7020	−536.7889	220	225	229	220.0463	CV F8	234.4399	221.4816
25	Poly (propylene)	0.6610	−104.2434	233	219	221	232.9725	CV F5	229.4755	232.6248
26	Poly (1-pentene)	0.8700	−303.4541	220	218	221	220.0052	CV F7	222.9140	220.2967
27	Poly (chlorotrifluoroethylene)	8.4682	−240.3014	373	336	332	372.9578	CV F7	333.3487	368.9975
28	Poly (1-hepatene)	2.0450	−802.8461	220	225	229	220.0160	CV F9	237.4576	221.7593
29	Poly (heptafluoropropyl ethylene)	8.5430	−602.6258	331	329	328	330.9970	CV F10	330.0105	330.8983
30	Poly (5-methyl-1-hexene)	1.3176	−644.5946	259	217	222	258.9642	CV F6	224.3646	255.5039
31	Poly (3-phenyl-1-propene)	8.0160	−707.0980	333	319	318	332.9710	CV F9	319.0645	331.5830
32	Poly (1,2-butadiene)	1.0067	−165.3425	269	223	225	268.9610	CV F4	227.4176	264.8066
33	Poly (ethyl methacrylate)	10.5208	−573.5125	324	360	357	324.0355	CV F10	359.1486	327.5471
34	Poly (isopropyl a-chloroacrylate)	10.6624	−763.5746	363	358	356	362.9920	CV F2	353.8745	362.0804
35	Poly (n-propyl a-chloroacrylate)	10.7744	−942.4947	344	356	355	344.0107	CV F6	354.9631	345.1059
36	Poly (isobutyl methacrylate)	10.0135	−1023.5389	337	342	342	337.0036	CV F3	340.7797	337.3814
37	Poly (neopentyl methacrylate)	8.2808	−1162.3670	306	312	314	306.0070	CV F8	312.4459	306.6511
38	Poly (2-ethylbutyl methacrylate)	6.1507	−1556.3205	284	271	275	283.9889	CV F2	273.3029	282.9202
39	Poly (n-hexyl methacrylate)	9.0823	−2310.6755	274	299	302	274.0253	CV F10	299.7605	276.5989
40	Poly (n-octyl methacrylate)	7.2768	−3875.9682	228	236	241	228.0096	CV F8	239.5428	229.1627
41	Poly (n-propyl methacrylate)	10.0625	−861.9543	308	347	346	308.0392	CV F1	346.0381	311.8392
42	Poly (isopropyl methacrylate)	10.0824	−704.7469	354	350	349	354.0024	CV F7	353.0622	353.9075
43	Poly (3,3-dimethylbutyl methacrylate)	10.2182	−1655.4510	318	331	333	318.0116	CV F4	329.6541	319.1760
44	Poly (sec-butyl methacrylate)	9.0868	−960.1252	330	329	330	329.9977	CV F10	329.2932	329.9274
45	Poly (phenyl methacrylate)	14.3442	−1124.6720	407	406	398	406.9925	CV F5	400.2030	406.3135
46	Poly (methyl methacrylate)	10.9884	−369.3694	378	372	367	377.9932	CV F6	373.6358	377.5567
47	Poly (t-butyl methacrylate)	10.0572	−819.0548	380	347	347	379.9624	CV F2	341.4221	376.1084
48	Poly (2-hydroxyethylmethacrylate)	13.3062	−696.3043	396	400	393	395.9980	CV F7	393.8416	395.7829
49	Poly (1-phenylethyl methacrylate)	6.8578	−1465.5384	299	284	287	298.9859	CV F4	284.7007	297.5573
50	Poly (ethyl a-chloroacrylate)	11.7166	−627.3794	366	377	373	366.0102	CV F3	376.9618	367.1050
51	Poly (sec-butyl a-chloroacrylate)	10.0080	−1051.9500	347	341	342	347.0035	CV F5	351.0209	347.4037
52	Poly (pentyl methacrylate)	8.3170	−1721.6799	268	300	304	268.0372	CV F9	301.7211	271.4057
53	Poly (trimethylsilyl methacrylate)	10.5548	−1000.3994	341	351	351	341.0078	CV F5	348.4749	341.7548
54	Poly (2,3-xylenyl methacrylate)	15.1894	−1527.7505	398	410	400	398.0048	CV F10	404.8272	398.6869
55	Poly (2,6-xylenyl methacrylate)	18.4630	−1353.6069	440	465	435	440.0134	CV F4	456.5018	441.6622
56	Poly (cyclobutyl methacrylate)	9.1436	−893.9148	351	332	332	350.9853	CV F10	340.3664	349.9223
57	Poly (cyclopenty methacrylate)	8.8336	−1094.4131	348	322	324	347.9728	CV F1	323.2202	345.4975
58	Poly (2-tert-butylaminoethyl methacrylat)	8.6144	−1712.1796	306	305	308	305.9889	CV F8	301.7353	305.5646
59	Poly (3,3,5-trimethylhexylmethacrylate)	7.0040	−2925.2411	274	253	258	273.9797	CV F1	256.9445	272.2758
60	Poly (2-decyl methacrylate)	5.1567	−4966.1840	208	178	197	207.9730	CV F3	165.8590	203.7628

Minimum		0.6610	−4966.1840	194	174	191	193.9905	–	165.8590	193.0971
Mean		7.6900	−1074.1949	307	305	305	306.8333	–	306.9183	306.8418
Median		8.2989	−810.9505	307	309	311	307.0231	–	313.8728	309.2452
Std		4.5090	974.1426	70	71	65	69.9740	–	67.2626	69.3736
Maximum		18.4630	−50.0178	440	465	435	440.0134	–	456.5018	441.6622
CC w. $T_{g}(K)$		89.78%	25.39%	–	95.10%	95.21%	99.99%	–	–	99.95%

Notes: Columns 2–4 contain Θ–the molecular traceless quadrupole moment and Φ–the molecular average hexadecapole moment for polymers. Column 5 contains the experimental target variable, $T_{g}(K)$–glass transition temperature. Columns 6 and 7 are estimated $T_{g}(K)$ from [48] and [49], respectively, that are based upon the QSPR (quantitative structure property relationship) and SVR (support vector regression). Column 10 contains the predicted $T_{g}(K)$ when an observation is utilized to carry out cross validation (CV). The relevant index (column 9), “CV F1,” “CV F2,” ..., and “CV F10,” means the *i*-th CV fold and the relevant models are shown in Table 2 as M2.CV F1, M2.CV F2, ..., and M2.CV F10. Columns 5 and 10 are plotted in Fig. 4. Columns 8 and 11 show $T_{g}(K)$ predicted from the model M1 and model M2 reported in Table 2. Fig. 3 plots their predictions with experimental $T_{g}(K)$ (column 5).

**Figure 3:**
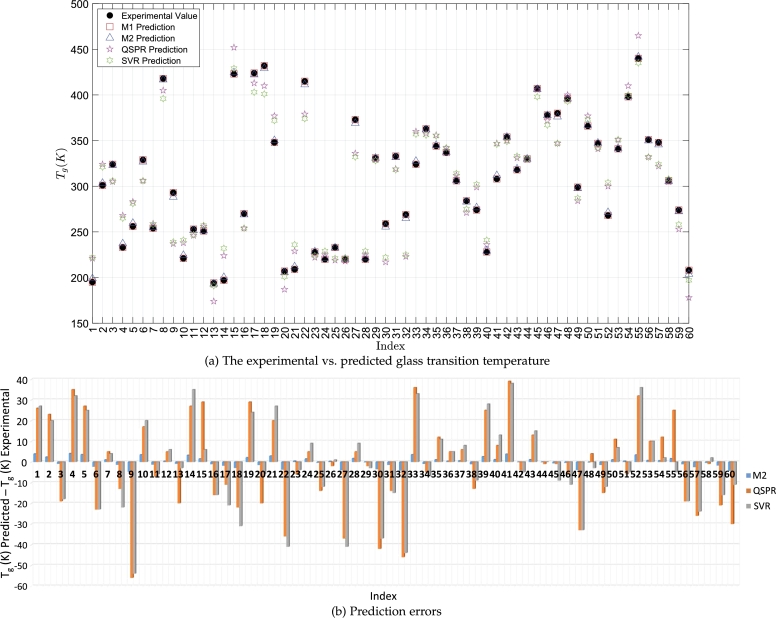
Experimental vs. predicted glass transition temperature (a) and prediction errors (b). Table 2 shows M2–our final model. M2's predicted values are reported in column 11 of Table 1. Table 2 also shows M1. M1's predicted values are reported in column 8 of Table 1. The legend “QSPR Prediction” is associated with column 6 in Table 1, which is from [48] based on the quantitative structure property relationship. The legend “SVR Prediction” is associate with column 7 in Table 1, which is from [49] based on the support vector regression.

In order to investigate the potential boundary of performance, we build Model M1 (see Table 2) from all available samples. Model M1's predicted results are provided in column 8 of Table 1 and plotted in Fig. 3. It results in *MAE*, *CC*, and RMSE of 0.0202 (0.01% of the mean of experimental $T_{g}(K)$), 99.99%, and 0.0245 (0.01% of the mean of experimental $T_{g}(K)$), respectively.

**Table 2:** Parameter estimates.

Model		Estimates					
		*σ*	*β*_0_	*β*_1_	*β*_2_	*σ*_*l*_	*σ*_*f*_
M1		0.7008	221.3984	14.1894	0.0226	7.2420	21.5963

M2	M2.CV F1	0.7154	221.6447	14.2088	0.0220	6.6682	21.0347
	M2.CV F2	0.6916	222.7540	13.9076	0.0224	10.3529	21.7934
	M2.CV F3	0.6895	225.9698	14.2142	0.0269	6.3166	21.4747
	M2.CV F4	0.6951	216.2863	14.5951	0.0216	6.4624	21.6913
	M2.CV F5	0.7045	222.5800	14.1322	0.0230	9.0863	21.7621
	M2.CV F6	0.7036	221.1307	14.3183	0.0232	8.0641	21.7162
	M2.CV F7	0.7093	217.1366	14.3862	0.0215	4.1938	20.7816
	M2.CV F8	0.6874	222.7193	13.8853	0.0217	4.1930	21.6106
	M2.CV F9	0.6909	224.0610	13.9405	0.0222	7.1492	21.0809
	M2.CV F10	0.7222	221.2657	14.3254	0.0223	6.4636	21.8385

Notes: $\beta_{0}$ is associated with the intercept, $\beta_{1}$ with Θ, and $\beta_{2}$ with Φ. M1 is constructed with all available samples. M2.CV F1, M2.CV F2, ..., and M2.CV F10 are constructed with samples marked with “CV F1,” “CV F2,” ..., and “CV F10.” We arrive at M2's predictions via applying M2.CV F1, M2.CV F2, ..., and M2.CV F10 to all available samples and calculating the mean of 10 predicted values for every sample. M1, M2.CV F1, M2.CV F2, ..., and M2.CV F10 are based upon the isotropic exponential kernel and linear basis with non-standardized independent variables.

## 4 Result and discussion

### 4.1 Accuracy

Model M2–the final model–is shown in Table 2. It reflects accurate matches between predictions and $T_{g}$ obtained experimentally. Detailed numerical results are reported in columns 5 and 11 of Table 1 and plotted in Fig. 3. The *MAE*, *CC*, and RMSE are 1.8373 (0.60% of the mean of experimental $T_{g}(K)$), 99.95%, and 2.2762 (0.74% of the mean of experimental $T_{g}(K)$), respectively. These suggest good performance and match Model M1's performance closely.

### 4.2 Stability

**Figure 4:**
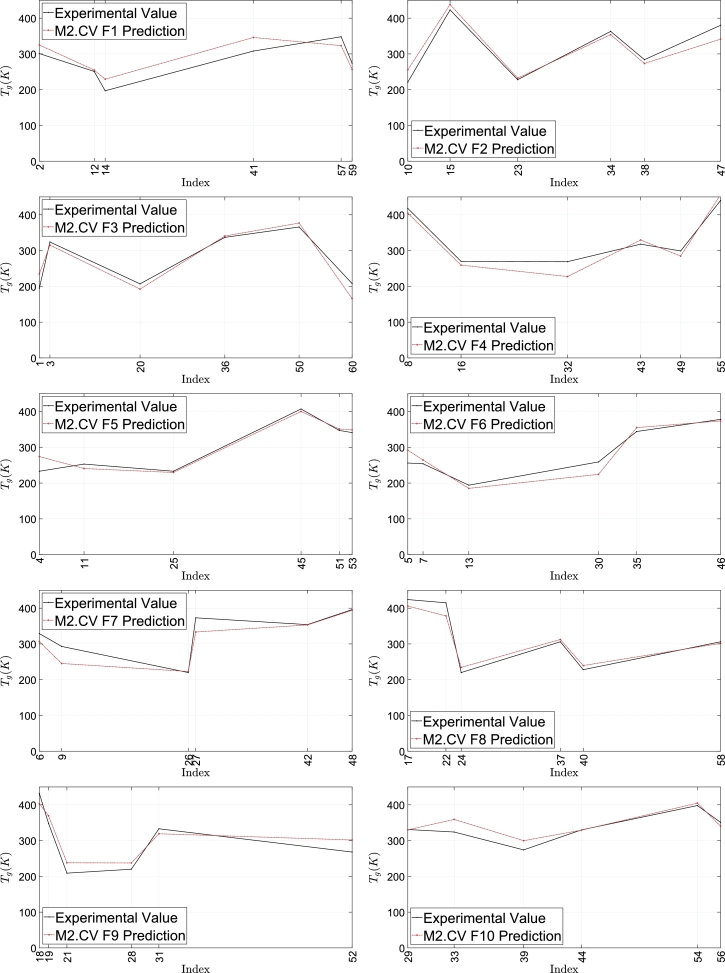
Cross validation. M2.CV F1, M2.CV F2, ..., and M2.CV F10 are reported in Table 2. Numerical results based on M2.CV F1, M2.CV F2, ..., and M2.CV F10 are shown in column 10 of Table 1.

From Table 2, one can see that parameter estimates are, in general, stable. To see implications of parameter estimates on stability of predictions, Table 3 provides performance details for all folds, whose associated predicted values are reported in column 10 of Table 1 and plotted in Fig. 4. One can see that all of the 10 folds, in general, have maintained stable and high CCs when switching from the training to the validation. Averagely, we have the MAE and RMSE as 6.00% and 7.34% of the validation samples' experimental mean, suggesting that errors beyond training are controllable. In order to cancel out idiosyncratic irregularities across different folds and build final more stable predictions, we construct M2 by making it a combined approach. In particular, Model M2's predictions are arrived at through applying M2.CV F1, M2.CV F2, ..., and M2.CV F10 to all available samples and calculating the mean of 10 predicted values for every sample. M2's performance is shown in Section 4.1, where one can see that it manifests high accuracy.

**Table 3:** Cross validation.

	Correlation coefficient		Root mean square error				Mean absolute error			
	Training	Validation	Training	% of Training Sample's Mean $T_{g}$	Validation	% of Validation Sample's Mean $T_{g}$	Training	% of Training Sample's Mean $T_{g}$	Validation	% of Validation Sample's Mean $T_{g}$
CV F1	99.99%	86.51%	0.026	0.01%	25.801	9.22%	0.021	0.01%	23.356	8.35%
CV F2	99.99%	95.65%	0.025	0.01%	22.748	7.19%	0.020	0.01%	18.654	5.89%
CV F3	99.99%	94.89%	0.024	0.01%	24.963	9.15%	0.019	0.01%	19.905	7.30%
CV F4	99.99%	98.06%	0.024	0.01%	20.954	6.24%	0.019	0.01%	18.012	5.37%
CV F5	99.99%	96.53%	0.024	0.01%	18.085	5.98%	0.020	0.01%	12.530	4.14%
CV F6	99.99%	94.67%	0.025	0.01%	21.667	7.72%	0.021	0.01%	17.539	6.25%
CV F7	99.99%	94.36%	0.025	0.01%	26.956	8.23%	0.021	0.01%	19.281	5.89%
CV F8	99.99%	99.39%	0.022	0.01%	18.591	5.87%	0.018	0.01%	15.242	4.82%
CV F9	99.99%	97.25%	0.024	0.01%	25.072	8.31%	0.020	0.01%	24.073	7.98%
CV F10	99.99%	89.96%	0.025	0.01%	18.530	5.54%	0.021	0.01%	13.344	3.99%

Minimum	99.99%	86.51%	0.022	0.01%	18.085	5.54%	0.018	0.01%	12.530	3.99%
Mean	99.99%	94.73%	0.024	0.01%	22.337	7.34%	0.020	0.01%	18.194	6.00%
Median	99.99%	95.27%	0.025	0.01%	22.207	7.45%	0.020	0.01%	18.333	5.89%
Maximum	99.99%	99.39%	0.026	0.01%	26.956	9.22%	0.021	0.01%	24.073	8.35%
Std	0.00%	3.85%	0.001	0.00%	3.280	1.38%	0.001	0.00%	3.805	1.51%

Notes: “CV F1,” “CV F2,”, ..., and “CV F10” mean the 1st, 2nd, ..., 10th CV fold. Predictions for “CV F1,” “CV F2,”, ..., and “CV F10” are produced by models M2.CV F1, M2.CV F2, ..., and M2.CV F10.

### 4.3 Comparisons

In Table 4, M2's performance is compared with that from two other models, QSPR (Quantitative Structure Property Relationship) [48] and SVR (Support Vector Regression) [49], in previous studies. One can see that for nearly all of the 60 samples, M2 produces predictions that are more accurate from the perspective of the absolute error. This is visualized in Fig. 3. The RMSE from M2 is 10.33% of the RMSE from QSPR [48] and 10.66% of the RMSE from SVR [49], and the MAE from M2 is 10.23% of the MAE from QSPR [48] and 10.82% of the MAE from SVR [49].

**Table 4:** Performance comparisons.

Model	Correlation coefficient	Root mean square error	% of Sample Mean *T*_*g*_	Mean absolute error	% of Sample Mean *T*_*g*_	M2 vs. QSPR	M2 vs. SVR
M2	99.95%	2.2762	0.74%	1.8373	0.60%	60/60 (100%)	59/60 (98.33%)
QSPR	95.10%	22.0348	7.18%	17.9667	5.86%	0/60 (0%)	–
SVR	95.21%	21.3499	6.96%	16.9833	5.54%	–	1/60 (1.67%)

Notes: Table 2 contains details of M1 and M2. Model QSPR (Quantitative Structure Property Relationship) is reported in [48]. Model SVR (Support Vector Regression) is reported in [49]. Column 7 compares M2 and QSPR's performance, where 60/60 (100%) reflects that M2 produces predicted values with absolute errors (AEs) smaller than AEs from QSPR's predicted values for 60 out of 60 samples. Column 8 makes comparisons between M2 and SVR's performance. Similarly, 59/60 (98.33%) reflects that M2 produces predicted values with AEs smaller than AEs from SVR's predicted values for 59 out of 60 samples.

## 5 Conclusion

We build the GPR model for predicting glass transition temperature, $T_{g}$, from Θ–the molecular traceless quadrupole moment and Φ–the molecular average hexadecapole moment for polymers. Our model manifests high stability and accuracy, which indicates the GPR's potential to understand and model relationships between fundamental quantum chemical parameters and $T_{g}$. This model is rather simple and needs fewer inputs than some other approaches. It can be applied to a variety of polymers with Tg values below or above room temperature.

## Declarations

### Author contribution statement

Yun Zhang: Conceived and designed the experiments; Analyzed and interpreted the data; Contributed reagents, materials, analysis tools or data; Wrote the paper.

Xiaojie Xu: Performed the experiments; Contributed reagents, materials, analysis tools or data; Wrote the paper.

### Funding statement

This research did not receive any specific grant from funding agencies in the public, commercial, or not-for-profit sectors.

### Competing interest statement

The authors declare no conflict of interest.

### Additional information

No additional information is available for this paper.
